# Multimodal CT Imaging Characteristics in Predicting Prognosis of Wake-Up Stroke

**DOI:** 10.3389/fneur.2021.702088

**Published:** 2021-11-15

**Authors:** Fan Yu, Xuesong Bai, Arman Sha, Miao Zhang, Yi Shan, Daode Guo, Adam A. Dmytriw, Qingfeng Ma, Liqun Jiao, Jie Lu

**Affiliations:** ^1^Department of Radiology and Nuclear Medicine, Xuanwu Hospital, Capital Medical University, Beijing, China; ^2^Beijing Key Laboratory of Magnetic Resonance Imaging and Brain Informatics, Beijing, China; ^3^Department of Neurosurgery, Xuanwu Hospital, Capital Medical University, Beijing, China; ^4^China International Neuroscience Institute (China-INI), Beijing, China; ^5^Neuroradiology & Neurointervention Service, Brigham and Women's Hospital and Harvard Medical School, Boston, MA, United States; ^6^Department of Neurology, Xuanwu Hospital, Capital Medical University, Beijing, China; ^7^Department of Interventional Neuroradiology, Xuanwu Hospital, Capital Medical University, Beijing, China

**Keywords:** wake-up stroke, ischemic penumbra, collateral circulation, prognostic value, CT perfusion (CTP)

## Abstract

**Background:** Multimodal CT imaging can evaluate cerebral hemodynamics and stroke etiology, playing an important role in predicting prognosis. This study aimed to summarize the comprehensive image characteristics of wake-up stroke (WUS), and to explore its value in prognostication.

**Methods:** WUS patients with anterior circulation large vessel occlusion were recruited into this prospective study. According to the 90-day modified Rankin Scale (mRS), all patients were divided into good outcome (mRS 0–2) or bad (mRS 3–6). Baseline clinical information, multimodal CT imaging characteristics including NECT ASPECTS, clot burden score (CBS), collateral score, volume of penumbra and ischemic core on perfusion were compared. Multivariate logistic regression analysis was further used to analyze predictive factors for good prognosis. Area under curve (AUC) was calculated from the receiver operating characteristic (ROC) curve to assess prognostic value.

**Results:** Forty WUS were analyzed in this study, with 20 (50%) achieving good outcome. Upon univariable analysis, the good outcome group demonstrated higher ASPECTS, higher CBS, higher rate of good collateral filling and lower penumbra volume when compared with the poor outcome group. Upon logistic regression analysis, poor outcome significantly correlated with penumbra volume (OR: 1.023, 95% CI = 1.003–1.043) and collateral score (OR: 0.140, 95% CI = 0.030–0.664). AUC was 0.715 for penumbra volume (95% CI, 0.550–0.846) and 0.825 for good collaterals (95% CI, 0.672–0.927) in predicting outcome.

**Conclusions:**Penumbra volume and collateral score are the most relevant baseline imaging characters in predicting outcome of WUS patients. These imaging characteristics might be instructive to treatment selection. As the small sample size of current study, further studies with larger sample size are needed to confirm these observations.

## Introduction

Stroke is the second-leading cause of global morbidity and mortality. The incidence rate of stroke reaches to up 13.7 million per year worldwide (1). The disability rate is as high as 70–80%, which seriously endangers the life and health of the general population, causing great burden to families and society (2). Wake-up stroke (WUS) accounts for 14–28% of all cases (3, 4). As WUS occurs during sleep and patients may only recognize stroke symptoms when they awaken in the morning, the duration from last time seen well to symptom recognition for WUS ranges from up to almost 7 to 14 h (5). Thus, WUS is traditionally classified into the late-time window, and patients need commonly to be evaluated by CT perfusion (CTP) in order to assess the volume of ischemic core on perfusion and penumbra according to the latest guidelines (6). Furthermore, the recent published meta-analysis by EXTEND, ECASS-4 and EPITHET investigators also highlights the value for perfusion imaging in wake up stroke patients (7).

However, only minor differences in clinical and imaging characteristics were shown between WUS patients and early-time window stroke patients (8–10). For early-time window stroke patients, NECT and CT angiography (CTA) are essential for quick evaluation of early ischemic changes and early detection of large vessel occlusion, while CTP is not necessarily recommended (6). Considering the particularity of WUS, it arguably should not be simply classified into neither late nor early time window stroke. It is both possible and necessary to put forward tailored imaging evaluation criteria for WUS.

Recently published studies proposed different imaging evaluation criteria for WUS (11–14). One study (11) suggested that WUS should be evaluated by using the ratio of ischemic penumbra to hypo-perfusion area >50% on CTP. While another study (12) used the ratio of infarct core to ischemic penumbra <50% on CTP as the imaging standard to evaluate WUS. However, it is still difficult to evaluate WUS as both studies used totally different imaging standards and did not analyze the relationship between imaging and prognosis. The other two studies analyzed the correlation of CTP and CTA with prognosis of WUS, respectively. One study (13) used CTP for WUS evaluation and showed that CTP core volume (β = 0.403, *p* = 0.000) could predict NIHSS at 7 days in a multivariate analysis. While another study (14) used CTA for WUS evaluation and found that collateral circulation condition graded by the ASITN/SIR was an independent influencing factor for prognosis of wake-up ischemic stroke patients at 3 months. Although other studies attempt to predict the prognosis of WUS by using ischemic core on perfusion volume and collateral circulation, a major weakness of both studies was using separate indexes from just a single modality of CTP or CTA instead of multimodality assessment. Multimodal CT imaging can provide comprehensive image characteristics which are important for predicting prognosis. Thus, it may be advantageous to explore the predictive value of multimodality CT for WUS prognosis.

This prospective study aims to describe the relationship between multimodal CT imaging characteristics and outcome of WUS, and explore the quantitative and qualitative imaging criteria for distinguishing WUS patients with different outcomes.

## Methods

### Patient Selection Criteria

All patients were prospectively and consecutively admitted at a single quaternary comprehensive stroke center from September 2018 to September 2019. NECT, CTP, and CTA were performed for acute ischemic stroke patients suspected to have large vessel occlusion. The inclusion criteria were: (i) patients with ischemic stroke awaken with neurological deficits and last time seen well over 6 h; (ii) patient pre-morbid functional independence as determined by modified Rankin Scale (mRS) 0–2; (iii) CTA showing large vessel occlusion in the anterior circulation from the intracranial internal carotid artery to the M2-branch of the middle cerebral artery. Patients with intracranial hemorrhage or stroke mimics on NECT, known renal dysfunction, contrast allergy, poor imaging quality and loss of clinical data were excluded. Baseline clinical characteristics such as age, sex, last time seen well, national institute of health stroke scale (NIHSS) scores at admission and comorbidities were collected. The decision to offer treatment to this subset of patients was determined following discussion between the stroke physicians and the interventional neuroradiologists. Factors taken into consideration were: (i) the Alberta Stroke Program Early CT Score (ASPECTS) on NECT 6–10; (ii) perfusion image demonstrating a CBF/CBV mismatch pattern; (iii) no other contraindications. For patients who underwent endovascular thrombectomy (EVT), modified Thrombolysis In Cerebral Infarction (mTICI) were collected as the end of the treatment. For patients who underwent conservative treatment, no reperfusion (mTICI 0) was recorded. For 90-day clinical outcome, this was assessed with mRS by the neurologist and interventional neuroradiologist in clinical follow up or by phone call. Good outcome was defined as mRS 0–2 and poor outcome as mRS 3–6.

All procedures performed in the studies involving human participants were following the ethical standards of the institutional and/or national research committee (LYS[2020]132).

### Imaging Protocol

NECT, CTP and CTA of the cervical and cerebral arteries were performed with a third-generation dual-source CT scanner (Siemens SOMATOM Force, Siemens Healthcare, Forchheim, Germany). NECT was acquired with 120 kV, 300–375 mAs at a slice thickness of 0.625 mm, and reconstructed at 5 mm. CTP was acquired with 70 kVp, 100 mAs, gantry rotation time of 0.5 s, collimation of 192 × 0.6 mm, and coverage in the z-axis of 114 mm. Forty ml of iodinated contrast material (ioversol 370 mg/ml) was injected at a rate of 6 ml/s *via* the antecubital vein, followed by flushing with 40 ml of saline at 6 ml/s, and scanning commenced 5 s after the injection. The dynamic perfusion scan consisted of 22 slices of images, each with a thickness of 5 mm. CTA was acquired with 90 kVp, 100 mAs, a matrix of 512 × 512, from aorta arch to vertex. Intravascular bolus injections of 50 ml of contrast medium and 50 ml of saline then administered at a rate of 5 ml/s in all patients, and scanning commenced 2 s after the monitor region of aorta trigger the threshold of 100 HU. The CTA scan was at a slice thickness of 0.625 mm and reconstructed at 5 mm.

### Imaging Evaluation

CTA and CTP raw data were transformed to the Syngo.via workstation (Siemens, version VB 2.0), then post-processed by “neuro vascular” and “neuro perfusion” software, respectively in order to obtain the maximum intensity projection (MIP), cerebral blood flow (CBF), cerebral blood volume (CBV), mean time to transit (MTT), time to peak (TTP), time to maximum of the residue function (Tmax) imaging. All imaging data were evaluated by two neuroradiologists (with 5 years of experience in neuroimaging) and discussed to reach an agreement as needed.

Early ischemic change of the brain was assessed by ASPECTS (15) on NECT. To quantify the hemodynamic changes on CTP, region of interest was drawn manually at the most significant slice and mirrored to the contralateral hemisphere on syngo.via workstation, and relative perfusion index, such as relative CBF (rCBF), relative CBV (rCBV) was calculated. Then, ischemic core on perfusion and penumbra volume were automatically extracted by defining an ischemic core on perfusion threshold of rCBF <30% while penumbra for Tmax more than 6 s according to previous publication (16). The clot burden score (CBS), and collateral filling were graded on CTA-MIP followed the criteria proposed in the previous articles (17).

### Statistical Analysis

Statistical analyses were performed using the SPSS software (Version 21 for Windows, SPSS, Armonk, NY, IBM Corp, USA). (i) The differences in categorical variables between the good outcome group and poor outcome group were analyzed separately by Chi-squared test or Fisher exact test. The differences in continuous variables were analyzed by independent-sample *t*-test or Mann-Whitney *U* test; (ii) After variables with significant differences were identified by above tests, Logistic regression analysis was used to explore predictive factors for good prognosis; (iii) Area under curve (AUC) was calculated from receiver operating characteristic (ROC) curve to check the efficiency of significant factors in (ii). In all tests, *p* < 0.05 was considered statistically significant.

## Results

One thousand six hundred ninety-nine patients came to the hospital with acute ischemic stroke symptom during study period. Among them, only 97 patients were wake-up stroke. Then, 77 wake-up stroke patients underwent multimodality CT imaging. Fifty-six wake-up stroke patients were found with occlusion of internal carotid artery or middle cerebral artery (M1 and M2 segment). Sixteen patients were excluded due to loss to follow-up (*n* = 8), poor imaging quality (*n* = 7) and loss of clinical data (*n* = 1). Ultimately, 40 patients were included in this study.

Good outcome defined as mRS 0–2 were seen in 20 (50%) patients. Severe disability (mRS 5) occurred in 3 (7.5%) patients and death (mRS 6) in 4 (10%) patients. No significant difference was observed in clinical parameters between good or poor outcome groups, except for NIHSS, (see Table 1).

**Table 1:** Patient characteristics (*n* = 40).

	**Study group *N* = 40**	**mRS 0–2 *N* = 20**	**mRS 3–6 *N* = 20**	***p-*value**
**Baseline parameters**				
Age, mean ± SD, y	60.12 ± 11.84	57.64 ± 10.65	65.50 ± 12.41	0.110
Male sex, *n* (%)	27 (67.5%)	13 (65.0%)	14 (70.0%)	0.741
NIHSS score, mean ± SD	11.00 ± 3.22	11.64 ± 2.65	16.00 ± 4.92	0.010
**Comorbidities**, ***n*** **(%)**				
Hypertension	19 (47.5%)	8 (40.0%)	11 (55.0%)	0.567
Atrial fibrillation	12 (30.0%)	6 (30.0%)	6 (30.0%)	0.941
Diabetes mellitus	15 (37.5%)	7 (35.0%)	8 (40.0%)	0.774
Last time seen well, mean ± SD, h	10.44 ± 4.15	9.16 ± 3.04	12.22 ± 4.96	0.074
EVT, *n* (%)	24 (60.0%)	14 (70.0%)	10 (50.0%)	0.523
Reperfusion, *n* (%)				0.200
unsuccessful reperfusion	17 (42.5%)	6 (30.0%)	11 (55.0%)	
Successful reperfusion	23 (57.5%)	14 (70.0%)	9 (45.0%)	

*EVT, endovascular thrombectomy; IQR, interquartile range; NIHSS, National Institutes of Health Stroke Scale, an acute ischemic stroke severity score ranging from 0 to 42: mild stroke = 1–4; moderate stroke = 5–15; moderate-severe stroke = 16–20; and severe stroke = 21–42; mTICI, modified Thrombolysis In Cerebral Infarction ranging from 0 to 3: 0/1/2a = unsuccessful reperfusion; 2b/2c/3 = successful reperfusion*.

**Figure 1:**
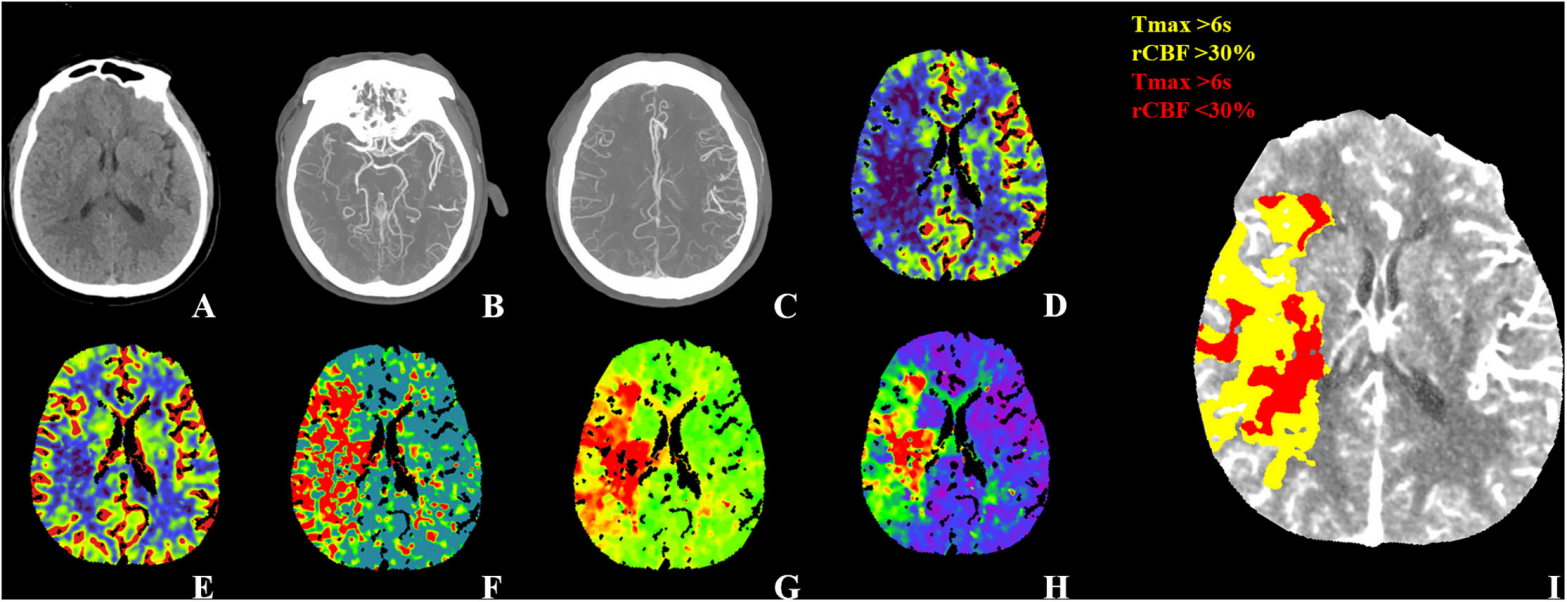
WUS with good outcome. Fifty-four-year-old male patient WUS patient last seen well 6.4 h before imaging. **(A)** The NECT didn't show any hypodensity with ASPECTS 10. **(B)** CTA-MIP showed right distal M1 segment occlusion with CBS 8. **(C)** CTA-MIP showed collateral filling nearly 100% of the MCA flow territory with collateral score three. The CTP showed **(D)** a large area with decreased CBF, **(E)** a smaller area with decreased CBV and **(F–H)** a large area with increased MTT, TTP, and Tmax. The CTP summary map **(I)** showed 76 ml of Tmax >6 s (yellow) and 34.93 ml of <30% rCBF decrease (red). The patient was treated EVT and showed good recovery with a 90-day mRS of one. WUS, wake-up stroke; ASPECTS, Alberta Stroke Program Early CT Score; MIP, maximum intensity projection; CBS, clot burden score; MCA, middle cerebral artery; EVT, endovascular thrombectomy; mTICI, modified Thrombolysis In Cerebral Infarction; mRS, modified Rankin Scale.

**Figure 2:**
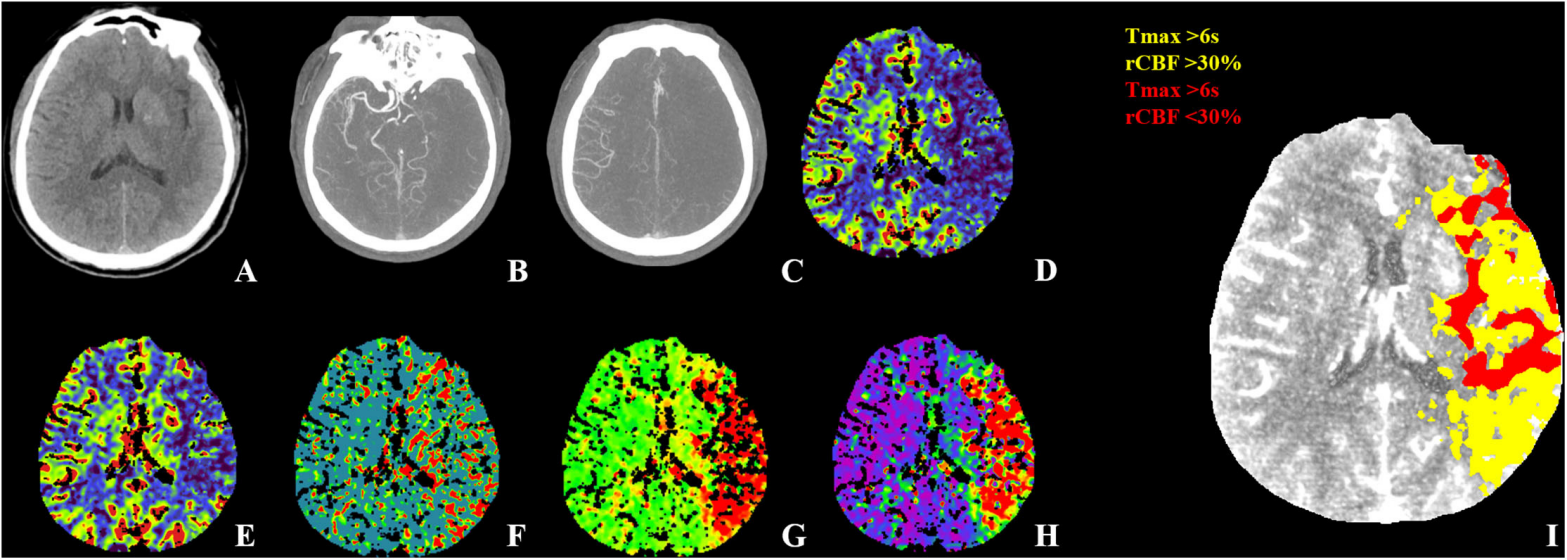
WUS with poor outcome. Fifty-seven-year-old male patient WUS patient last seen well 6.7 h before imaging. **(A)** The NECT show hypodensity in left the insular, lenticular nucleus, M1 and M2 with ASPECTS 6. **(B)** The CTA-MIP showed left ICA, M1 and M2 occlusion with CBS 0. **(C)** CTA-MIP showed collateral filling nearly 0% of the MCA flow territory with collateral score one. The CTP showed **(D)** a large area with decreased CBF, **(E)** CBV and **(F–H)** a large area with increased MTT, TTP, and Tmax. The CTP summary map **(I)** showed 93.73 ml of Tmax >6 s (yellow) and 59.08 ml of <30% rCBF decrease (red). The patient was treated EVT (mTICI 2b) and showed poor recovery with a 90-day mRS of six. WUS, wake-up stroke; ASPECTS, Alberta Stroke Program Early CT Score; MIP, maximum intensity projection; CBS, clot burden score; MCA, middle cerebral artery; EVT, endovascular thrombectomy; mTICI, modified Thrombolysis In Cerebral Infarction; mRS, modified Rankin Scale.

On NECT, ASPECTS was significantly higher in the good outcome group compared with poor [9 (8–10) vs. 8 (6–9), *p* = 0.017]. For CTP, perfusion abnormalities were observed in all WUS patients with decreased CBF, CBV and prolonged MTT, TTP as well as Tmax. However, no significant difference was observed in preliminary quantitative measure of perfusion parameters. Further quantitative measure showed similar ischemic core on perfusion volume between groups [24.64 ml (10.28–35.37) vs. 36.32 ml (20.66–114.28), *p* = 0.094] while significant larger penumbra volume in poor outcome group compared with good (119.81 ± 69.13 vs. 85.21 ± 48.35 ml, *p*= 0.020). On CTA, 11 cases with intracranial internal carotid artery occlusion while 29 cases with middle cerebral artery occlusion were found among 40 wake-up stroke patients. There was no significant difference in occlusion site between group (χ^2^ = 3.135, *p* = 0.077). The mean CBS was significant lower in poor outcome group (4.00 ± 3.56 vs. 7.35 ± 1.57, *p* = 0.003) with a lower rate of good collateral filling (20 vs. 85%, *p* < 0.001), (see Table 2; Figures 1, 2).

**Table 2:** Imaging characteristics (*n* = 40).

	**Study group** ***N* = 40**	**mRS 0–2** ***N* = 20**	**mRS 3–6 *N* = 20**	***p-*value**
**Baseline parameters**				
ASPECTS 0–10, median (IQR)	9 (7–10)	9 (8–10)	8 (6–9)	0.017
Occlusion site, *n* (%) ICA	11 (27.5%)	3 (15%)	8 (40%)	0.077
MCA	29 (72.5%)	17 (85%)	12 (60%)	
CBS, mean ± SD	5.75 ± 3.13	7.35 ± 1.57	4.00 ± 3.56	0.003
Good collateral, *n* (%)	21 (52.5%)	17 (85%)	4 (20%)	<0.001
Ischemic core on perfusion,	29.68	24.64	36.32	0.094
Median (IQR), ml	(10.63–54.84)	(10.28–35.37)	(20.66–114.28)	
Penumbra, mean ± SD, mL	117.99 ± 73.15	85.21 ± 48.35	119.81 ± 69.13	0.020
Ischemic tissue to infarct core,	4.65	4.55	4.80	0.850
Median (IQR)	(2.47–6.74)	(2.99–6.13)	(2.53–8.45)	
rCBF, mean ± SD	29.19 ± 11.82	30.32 ± 13.85	29.10 ± 9.51	0.555
rCBV, mean ± SD	40.12 ± 17.23	43.64 ± 17.89	38.41 ± 16.10	0.200
rMTT, mean ± SD	143.06 ± 56.76	152.28 ± 67.12	135.85 ± 44.55	0.310
rTTP, mean ± SD	151.77 ± 24.64	152.06 ± 28.20	151.99 ± 21.82	0.941
rTmax, mean ± SD	448.12 ± 285.03	503.57 ± 372.93	386.41 ± 150.30	0.223

*ASPECTS, Alberta Stroke Program Early CT Score; CT, computerized tomography; CBS, Clot Burden Score; rCBF, relative cerebral blood flow; rCBV, relative cerebral blood volume; rMTT, relative mean transit time; rTTP, relative time to peak; rTmax, time to maximum of the residue function; Good collateral = 2–3 score*.

**Figure 3:**
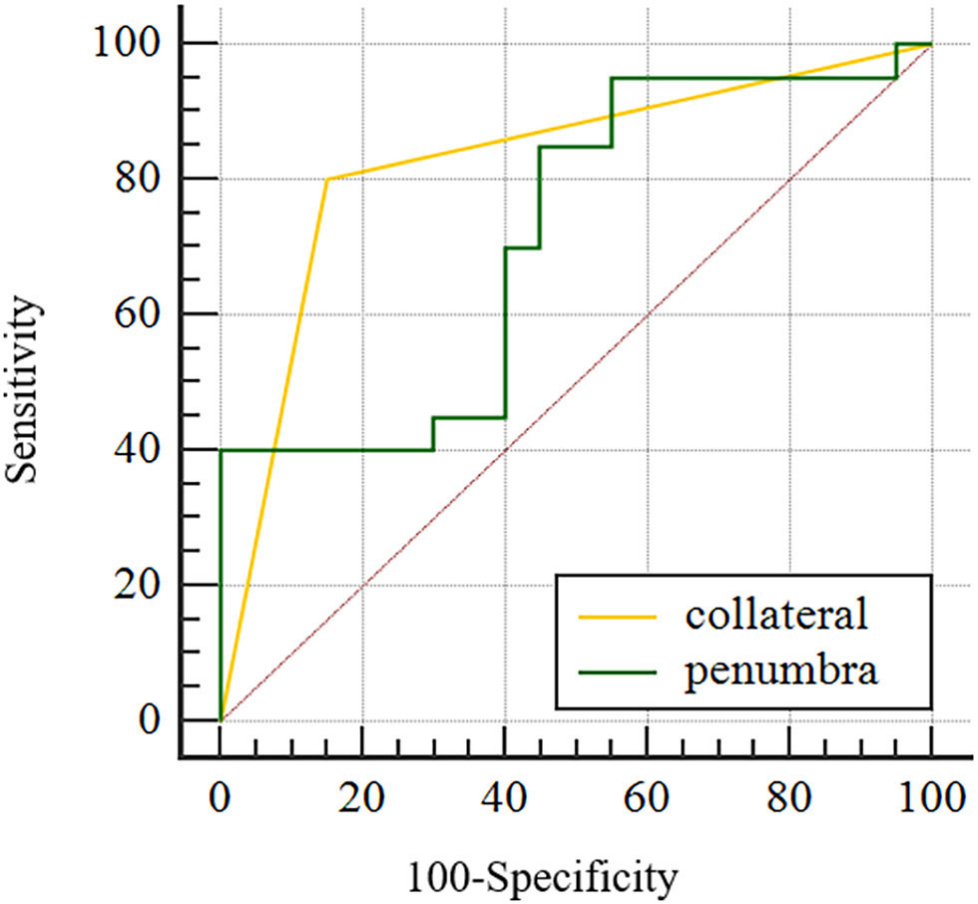
Area under the curve for good collateral and penumbra volume in predicting functional independence (mRS 0–2) for WUS with large vessel occlusion in the anterior cerebral circulation.

After subjecting all variables with significant differences identified by the above tests to logistic regression analysis, only penumbra volume (OR: 1.023, 95% CI = 1.003–1.043) and collateral score (OR: 0.140, 95% CI = 0.030–0.664) were found to be predictive for 90-day poor outcome, (see Table 3). AUC was 0.715 for penumbra volume (95% CI, 0.550–0.846) and 0.825 for good collaterals (95% CI, 0.672–0.927) in predicting outcome (Figure 3). At a cut-off value of 64.73, penumbra showed high sensitivity (0.95) and low specificity (0.45). At a cut-off value of one, collaterals showed high sensitivity (0.80) and high specificity (0.85).

**Table 3:** Predictors of patient outcome.

	**Odds ratio (OR)**	**95% Confidence intervals (CI)**	***p*-value**
ASPECTS 0–10	0.482	0.140-1.662	0.248
CBS	0.728	0.466-1.139	0.165
Collateral score	0.140	0.030-0.664	0.013^*^
Penumbra volume	1.023	1.003-1.043	0.024^*^

*ASPECTS, Alberta Stroke Program Early CT Score; CBS, Clot Burden Score*.

* *p-value of ≤0.05 was considered statistically significant*.

## Discussion

In this prospective study, comprehensive image characteristics were summarized by using multimodality CT in WUS patients. Furthermore, the relationship between imaging and WUS patients' outcome was also analysis. We found that baseline NECT ASPECTS, clot burden score and collateral score were significantly higher in good outcome group compared with poor while penumbra volume was lower. Further analysis demonstrated that penumbra volume and collateral score are likely the most relevant baseline imaging characters in predicting outcome of WUS patients.

Compensating collateral flow is critical in maintaining blood flow to the benign oligemia and penumbra and restraining evolution of brain ischemia in patients with large vessel occlusion. Previous meta-analysis showed that patients with better collateral circulation could have a smaller ischemic core on perfusion and less severe neurological symptoms at baseline and have a better chance of achieving a favorable or excellent functional outcome at 3 or 6 months after the index stroke (18). Another study (14) used CTA for WUS evaluation and found that collateral circulation condition graded by the ASITN/SIR was an independent risk factor for bad prognosis of wake-up ischemic stroke patients in 3 months. In this study, we found good collateral circulation (2–3 score) was a strong predictor for good outcome in WUS with relatively larger AUC than the penumbra volume. One multicenter study also showed that infarct growth was higher while penumbra salvage was lower among those with poor collaterals vs. those with good collaterals (19). If penumbra truly represents salvageable brain as is commonly maintained, collateral circulation appears to be a crucial the modulator of its fate.

Ischemic penumbra, indicating electrically non-functioning but metabolically viable brain tissue that is salvageable with rapid cerebral blood flow restoration, is the most common characteristic to measure ischemic range (20). CTP is the most common *in vivo* imaging to depict its “misery perfusion” in an emergent scenario. In this study, we found a larger penumbra volume and higher NIHSS score in poor outcome WUS patients. This finding seems contradictory to the commonly held concept that larger the salvable brain tissue volume, better the patient's outcome. However, we propose that this is explicable by the hypothesis that a larger penumbra represents a faster rate of progression from the hypo-perfused tissue to completed infarct. This characteristic will lead to larger final infarct volume as well as comparatively poor outcome. However, this hypothesis requires further confirmation, for instance by calculating the early infarct growth rate or analyzing the follow-up infarct volume on multisequence MRI. One another hand, if, for example, the penumbral tissue was located in a less eloquent part of the brain and the infarct core dominated the relationship to outcome, salvage of the penumbra would be less helpful. Supportive evidences include a meta-analysis by the HERMES collaborators (21) and a study which set up a model to predict outcome by collecting 1,476 ischemic patients (22). Both of them failed to find the associated between penumbra with either functional independence or functional improvement. The relationship between the penumbra and the infarct core may become more complex and is still under explosion.

For WUS patients, the good outcome group demonstrated higher NECT ASPECTS and higher CBS when compared with poor outcome group, which is largely consistent with previous studies focusing on onset clear stroke (23–25). However, NECT ASPECTS and CBS failed to be relevant with outcome upon logistic regression in this study. This could relate to the subjective evaluation method, lower specificity due to rater dependence, and lower sensitivity of NECT in detecting ischemic changes. In this study, we also evaluated relative perfusion parameters in predicting WUS patient outcome and failed to observe a significant difference. This result suggests in our view that it is the ischemic range, instead of the ischemic degree, which matters most for prognostication. However, this finding requires further confirmation.

There are some limitations of this study. Firstly, the sample size is moderate, and loss to follow-up rate is relatively high. Future studied with larger sample size are needed. Secondly, the post-processing software used for calculating penumbra and infarct core is not always consistent among studies. However, previous research has showed excellent agreement between RAPID software and syngo.via default settings (26). Thirdly, some other variables, such as sub-acute stroke complications, may also be valuable in predicting the prognosis of patients and are needed to be explored in future studies.

## Conclusion

NECT ASPECTS, clot burden score, penumbra volume and collateral score differ between good and poor outcome patients following WUS. Penumbra volume and collateral compensation are the most relevant baseline imaging characters in predicting outcome of WUS patients. These imaging characteristics might be instructive to treatment selection. Further studies with larger sample size are needed to confirm these observations.

## Data Availability Statement

The raw data supporting the conclusions of this article will be made available by the authors, without undue reservation.

## Ethics Statement

The studies involving human participants were reviewed and approved by Xuanwu Hospital, Capital Medical University. The patients/participants provided their written informed consent to participate in this study.

## Author Contributions

FY, XB, LJ, QM, and JL contributed to conception and design of the study. FY organized the database and wrote the first draft of the manuscript. XB performed the statistical analysis. XB, YS, AD, and MZ wrote sections of the manuscript. AS and DG performed imaging examination for patients. All authors contributed to manuscript revision, read, and approved the submitted version.

## Funding

This work was supported by Beijing Natural Science Foundation (Z190014).

## Conflict of Interest

The authors declare that the research was conducted in the absence of any commercial or financial relationships that could be construed as a potential conflict of interest.

## Publisher's Note

All claims expressed in this article are solely those of the authors and do not necessarily represent those of their affiliated organizations, or those of the publisher, the editors and the reviewers. Any product that may be evaluated in this article, or claim that may be made by its manufacturer, is not guaranteed or endorsed by the publisher.

## Abbreviations

WUS: wake-up stroke
CTP: CT perfusion
CTA: CT angiography
mRS: modified Rankin Scale
NIHSS: national institute of health stroke scale
EVT: endovascular thrombectomy
mTICI: modified Thrombolysis In Cerebral Infarction
MIP: maximum intensity projection
CBF: cerebral blood flow
CBV: cerebral blood volume
MTT: mean time to transit
TTP: time to peak
Tmax: time to maximum of the residue function
CBS: clot burden score
AUC: Area under curve
ROC: receiver operating characteristic.
